# Silent Prions and Covert Prion Transmission

**DOI:** 10.1371/journal.ppat.1005249

**Published:** 2015-12-10

**Authors:** Candace K. Mathiason

**Affiliations:** Prion Research Center, Department of Microbiology, Immunology, and Pathology, College of Veterinary Medicine and Biomedical Sciences, Colorado State University, Fort Collins, Colorado, United States of America; Washington University School of Medicine, UNITED STATES

Prions are infectious agents with zoonotic potential that cause progressive neurodegenerative diseases, known as the transmissible spongiform encephalopathies (TSEs), in animals and humans. Prion disease can be initiated by a spontaneous event, handed down within families from generation to generation through genetic inheritance, or transmitted between susceptible hosts via direct exchange of bodily fluids and/or excreta (saliva, blood, urine, and feces), indirect contact with contaminated environments (bedding, buckets, soils, water), or during medical intervention (transfusion, dura mater grafts, human growth hormone, contaminated instruments) [1–3]. Observation and study of prion diseases has led to an understanding that transmission dynamics and efficiency varies among prions, dependent upon agent strain, inoculation route, and host factors/genetics [1,2].

A hallmark of prion diseases is a protracted incubation period. In fact, the extended incubation times associated with these diseases led early researchers to investigate a “slow” or lentiviral etiology [4,5]—viruses with a known lengthy asymptomatic phase of disease. It was only after seminal experiments demonstrated that infectivity was primarily associated with protein and not nucleic acid that the term “prion” (proteinacious infectious particle) was coined to classify these diseases [6].

Extensive mapping of prion deposition and trafficking in conjunction with new assays with increased detection sensitivity have enhanced our understanding of the long silent phase of prion disease. While titer and timing may vary with the strain and route of inoculation, prions are initially detected in the lymphoreticular system (variant Creutzfeldt-Jakob disease [vCJD], scrapie, transmissible mink encephalopathy [TME], bovine spongiform encephalopathy [BSE], chronic wasting disease [CWD]) [1,2]. Of particular interest is the finding that prions replicate in the spleen of infected hosts that do not develop clinical disease [7–9]. It has been postulated that these individuals maintain splenic prion titers without progression to the central nervous system—i.e., incubation time exceeds the natural lifespan of the host (Fig 1 derived from [7]). Thereby creating a population of individuals that harbor prions and may be a potential source of infection for the duration of their lives—i.e., silent carriers.

**Fig 1:**
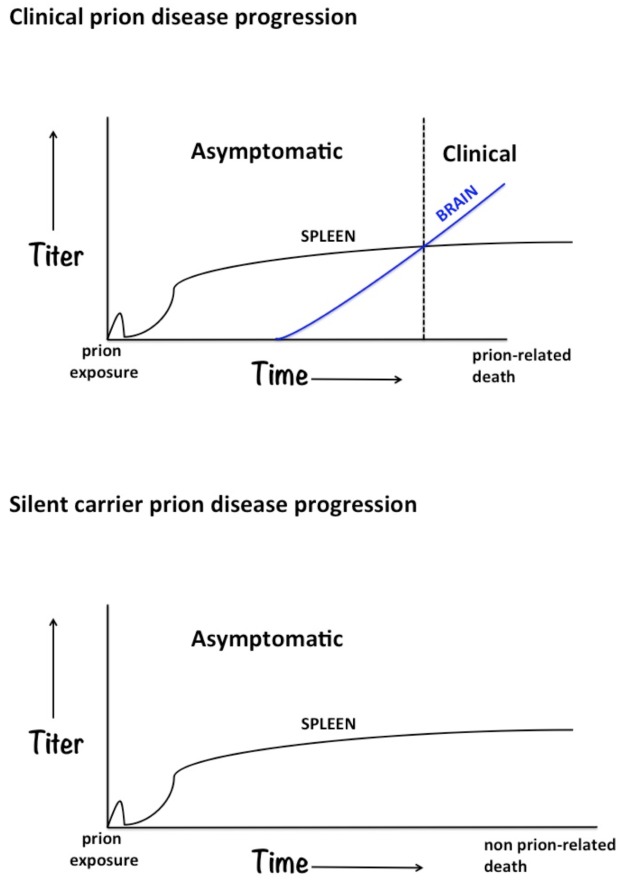
Clinical and silent carrier prion disease progressions.

## What Causes Prion Disease?

The misfolding and aggregation of a highly conserved cellular protein, the prion protein (PrP^C^), which is shared by all mammalian species and is encoded by the PRNP gene [10].

## What Makes Prion Diseases Unique?

The ability of this misfolded protein, without the classical aid of nucleic acid, to act as an infectious agent with the capacity to be transmitted from one susceptible host to another [6].

## What Are the Potential Outcomes of Prion Exposure?

Prion diseases are often described as invariably fatal. However, upon prion exposure, it is possible that the threshold necessary to initiate disease will not be crossed and neither infection nor disease will ensue. A second potential outcome is that susceptible hosts will be exposed to sufficient infectious prions to initiate clinical disease progression with neurologic presentation, resulting in the inevitable death of the host. The best examples of this include the BSE, or mad cow disease, outbreak in cattle and the subsequent human outbreak of a new or variant form of Creutzfeldt-Jakob disease (CJD), vCJD, which was later linked to BSE transmission via contaminated products [1]. Other known prion diseases with fatal outcomes include sheep and goat scrapie, CWD in cervid species, and perhaps the most notorious prion disease, that endemic among the Fore tribe of Papua New Guinea—kuru [1,2]. For some hosts infected with these TSEs, the asymptomatic phase of disease may persist throughout their entire lifespan, allowing the host to escape obvious clinical presentation and associated death, representing the third potential outcome of prion exposure—the silent carrier state.

## What Is Known about Transmissible Prion Diseases and the Silent Carrier State?

Demonstration of prion transmission during the protracted silent carrier state of disease has been witnessed in humans post-blood product distribution, tissue grafting, and human growth hormone use and has been investigated experimentally in animals [1,2]. The full implications of this silent carrier state remains largely unresolved because of the length of time required to observe associated outcomes.

### Human prion disease

A retrospective analysis of lymphoid tissues in the United Kingdom, where the BSE and subsequent vCJD outbreaks were most prominent, revealed aberrant prion deposition in approximately 1 in 2,000 individuals, suggesting that these individuals may be silent prion carriers [11]. Likewise, kuru can have a silent carrier state for up to 50 years [12], extended by certain polymorphisms in the prion protein [1,2], lending an air of uncertainty to the fate of silent carriers.

Contemporary in vitro assays with the ability to detect minute quantities of prions, i.e., protein misfolding cyclic amplification (PMCA) and real-time quaking-induced conversion (RT-QuIC), have detected prions in saliva, urine, and feces of vCJD-infected humans [13]. To date, vCJD infectivity has not been conveyed by these bodily secretion and/or excretions when assessed by the most sensitive test, bioassay. Conversely, vCJD disease transmission has been demonstrated via blood and tissues derived from asymptomatic donors as well as the surgical instruments used to harvest these products [14]. Consequently, there is increasing awareness to the potential that some individuals may harbor lifelong covert TSE infection and may unwittingly contribute to continued prion dissemination.

Utilization of bioassay in the native host and the highly sensitive in vitro detection methodologies mentioned above have provided evidence for a silent carrier state in animal prion diseases.

### Scrapie

Scrapie infections in sheep and goats have been recognized for at least 200 years [2]. The clinical disease has been transmitted by direct contact between infected and susceptible animals, presumably because of exposure to saliva, urine, feces, [2] or blood [15], all of which have recently been demonstrated to contain infectious prions. Historical reports recognized a cyclical increase in scrapie incidence during the lambing season [16]. Bioassay in the native host has confirmed that sufficient infectious prions are present in reproductive fluids and tissues (milk, placenta, and gestational fetal tissues) to help explain this phenomenon [2]. Prions associated with scrapie can persist in the environment for up to 16 years and readily infect sheep and goats pastured on lands that previously housed scrapie-infected animals [17]. Transmission occurs in healthy-looking herds [16].

### Chronic wasting disease

CWD, the prion disease of cervids, has been documented in farmed and free-range populations [2]. The disease is characterized by remarkably high prion replication in host tissues that are readily shed in bodily fluids and excretions (saliva, blood, urine, feces), [2] permitting efficient horizontal transmission by direct animal-to-animal contact and via indirect contact with contaminated environments [2]. In addition, recent studies provide evidence for mother-to-offspring prion dissemination [2]. CWD prions are harbored and shed by infected hosts throughout the disease course—minutes post-exposure to terminal-stage disease—[18,19] and can persist in the environment for at least three years [2].

### Bovine spongiform encephalopathy

BSE is the only prion known to have crossed the species barrier under natural conditions, infecting humans, felids, and ungulates [2]. While BSE has a more limited lymphoreticular association than vCJD, scrapie, or CWD [1,2], prions are readily transmitted via blood transfusion during clinical and subclinical infection [15]. Prions have been detected by PMCA in saliva from clinical and preclinical cattle [20], yet to date infectious BSE prions have not been detected in saliva, urine, or feces. Of growing concern is the knowledge that prions sourced from silent carriers are capable of disease transmission and progression for all TSEs—CJD, scrapie, CWD, and BSE.

## What Is Covert Prion Transmission?

The introduction of sufficient prions—in tissues, bodily fluids, or excretions shared and shed into the environment or transferred via medical intervention—during the asymptomatic phase of disease to transmit and perpetuate prion infection in susceptible hosts coming into contact with them.

## Why Are Silent Prion Infections and Covert Prion Transmission Important?

Conversion-competent prions have been shown to traverse mucosal surfaces finding their way into the blood within minutes of prion exposure, where they are ferried throughout the host for the duration of disease [18]. This suggests that all parts of a prion-infected host contain prions—early and persistent. Prion-infected hosts, while remaining healthy and reproductively competent for a long time—perhaps even their entire life span—harbor prions in blood, saliva, urine, feces, and the reproductive milieu that could be shared between individuals or shed into the environment. Prions are thus available for consumption by the same susceptible species or by potential reservoir species sympatric with prion-harboring hosts. We know infectious prions from asymptomatic scrapie, CWD, BSE, and CJD-infected hosts can be transmitted by blood products or tissues to cause disease in recipients. Of additional concern is the potential for prions to cross the species barrier into reservoir hosts or humans. BSE, while not known for efficient transmission among cattle, has crossed the species barrier to infect humans (vCJD), felids (FSE), and ungulate species [2]. Is this scenario a concern for CWD, scrapie, and BSE? Do new prion strains with altered tropism exist that pose an enhanced risk for disease transmission during the silent carrier state? To this end, continued study of prions during this silent carrier state will enhance our understanding of prion pathogenesis, transmission, and development of therapeutics and vaccines to mitigate their spread.
